# Effect of Short-term Exercise on Appetite, Energy Intake and Energy-regulating Hormones 

**Published:** 2013-07

**Authors:** Mohsen Ebrahimi, Farhad Rahmani- Nia, Arsalan Damirchi, Bahman Mirzaie, Sepide Asghar Pur

**Affiliations:** 1 Department of Physical Education & Sport Science, University of Semnan, Semnan, Iran; 2 Department of Sport Science, University of Guilan, Rasht, Iran

**Keywords:** Aerobic exercise, Appetite, Appetite-regulating hormone, Energy intake

## Abstract

***Objective(s)*** ***:*** The purpose of this study was to investigate effects of short-term aerobic exercise on energy intake, appetite and energy-regulating hormones in free-living men and women.

***Materials and Methods:*** Sixteen (eight men, eight women) sedentary young normal weight subjects participated in two experimental conditions with two days apart: five days control with no exercise, and five days exercise (55% MHRR for 45 min/day). Subjects recorded dietary intake using a food diary and self-weighed intake during each five days. Appetite questionnaire (visual analogue scale) was completed each morning in the fasted state. Blood samples were taken in the morning on the 6th day in fasting status after control and exercise conditions.

***Results:*** No significant changes were found in absolute energy intake, appetite rate and level of acylated ghrelin and leptin between conditions in both sexes. In women, insulin concentration decreased significantly after exercise. Relative energy intake was significantly lower after exercise in men. On average, women compensated for about 23% of the exercise-induced energy deficit but men did not (-10%).

***Conclusion:*** Our findings show that low-intensity exercise for five consecutive days cannot create a negative energy balance in women. It seems that women are more resistant to exercise-induced energy deficit.

## Introduction

Body weight is regulated through the balance between energy intake and energy expenditure. For weight control, many researchers and scientists recommend regular exercise in order to increase energy expenditure. In addition, recent studies show that exercise can modify energy intake through the adjustment of the energy-regulating hormones indirectly (1).

There is some evidence indicating that physical activity, as a tool for weight loss is more effective in men than women. Donnelly *et al* (2) and Potteiger *et- al* (3) showed that 16 months aerobic exercise decreased body fat in men with no significant effect in women consuming *ad libitum* diets. 

It was also reported that short-term exercise did not lead to compensation for energy intake over seven days in men (4), but increased energy intake in women (5). Similarly, women were shown to increase their energy consumption after an intensive bout of exercise (6) while men were not (7, 8).

The outcome of sex differences on the effect of exercise on subsequent energy intake and reducing body fat may be related to hormonal differences between men and women. Hickey *et al* (9) found that serum insulin and leptin after 12 weeks of exercise decreased in women. However, significant changes were not observed in the level of these two hormones in men. Hagobian *et al* (10) found that, in women, exercise altered energy-regulating hormones and increased energy intake, regardless of energy status. Nevertheless, in men, the response to exercise was abolished when energy balance was maintained. They suggested that the mechanisms to maintain body fat are more effective in women.

However, there are studies that reported contrary results. Interestingly, Staten (11) reported that energy intake after five days of intense exercise, significantly increased compared with five controls days (without exercise) in men, but there was no significant change in energy intake in women. Furthermore, Whybrow *et al* (12) found similar results after 14 days of exercise. 

**Table 1:** Characteristics of the subjects participated in the exercise

	Men	Women
Age (year)	21.63±1.76	21.25±1.38
Height (cm)	171.50±5.42	158.62±3.20
Weight (kg)	64.51±5.35	56.47±3.83
BMI (kg/m^2^)	22.22±1.60	22.41±1.55
Body fat (%)	19.00±3.38	28.68±1.92

Data expressed as mean±SD

**Table 2:** Characteristics of exercise

	Men	Women
Mean HR during exercise (beat/min)	140.80±2.82	141.35±3.58
% HRmax (%)	55.41±0.58	54.91±2.78
Treadmill speed (km/hr)	6.03±0.68	5.16±0.68
Exercise duration per day (min)	45	45
Exercise energy expenditure (kcal)	291.02±32.42	220.41±30.03

Data expressed as mean±SD

The reasons of those inconsistencies are not clear. However, research in this area is rather limited and this question remains unanswered that “are there sex differences in Energy-Regulating hormones, perceived appetite and *ad libitum *food intake in response to exercise?” For better understanding the effect of exercise on the hormonal regulation of appetite and food intake, it is important to examine all three as dependent variables (hormones, appetite, and food intake) in the same study. However, no published studies have compared men and women regarding all 3 variables. We hypothesized that if there are any sex differences, we can observe it in at least one of 3 variables. Thus, the present study aimed to identify the effect of short-term exercise on appetite, energy intake and some energy-regulating hormones in men and women.

## Materials and Methods

***Subjects***

Subjects were recruited from students in University of Guilan. Eight men and eight women were selected between volunteer according to our research criteria. All volunteers were healthy, non-smokers, sedentary (<1 hr/wk of regular exercise) and weight stable for the previous six month (<±2 kg) as determined by a health-history questionnaire. Selected women had regular menstrual cycles and were not on oral contraceptives. All subjects submitted written informed consents to participate and the study was approved by the local Committee of Ethics. Baseline characteristic of subjects can be seen in Table 1. 

***Experimental design***

Each subject was studied in two experimental conditions: five days control with no exercise, and five days exercise. The two conditions were separated by two days. In control condition, subjects were asked to refrain from any sports activity and heavy work. In exercise condition, subjects performed running on the treadmill for five consecutive days. Protocol design was randomized counterbalance and crossover. Protocol for women was started six days after menstruation. 

***Exercise protocol***

Subjects performed running on a treadmill for 45 minutes per day. The exercise intensity was at 55% of each subject’s estimated maximum heart rate reserve (MHRR) as previously described (13). Heart rate was measured continuously using a heart rate monitor (Polar F11 GRY, Finland). Heart rate and treadmill speed were recorded each 5 minutes during exercise for calculating average values. Exercise energy expenditure was calculated as energy expended above resting metabolic rate using the American College of Sports Medicine (ASCM) running equation (14) as previously described (15). Characteristics of exercise can be seen in Table 2.

***Food intake measurement***

Subjects recorded dietary intake using a food diary and self-weighed intake during each five days. Food intake was *ad libitum*. Prior to the study, subjects were instructed on how to record their food and drink intake accurately. All of subjects were using the University dining hall. Since the University has established a weekly meal plan, types of food intake were similar in both conditions. Food and drink intake were recorded using a set of digital weighing scales (Doulton Model: EK9150) and a self-record diary. The scales’ capacity was 1 to 5000 g. Also, subjects weighed back leftovers. If possible, food labels and packets were also held and delivered to the investigator. For foods and fluids eaten away from the dining hall, subjects were instructed to report the type and amount of food as exactly as possible in the food diary. Subject’s diary sheet was checked every night by investigator for demystification. Furthermore, the subjects were able to make phone calls to the investigator and ask questions during the study. Energy intake was calculated via Nutrition IV software (adjusted to the Iranian people diet).

**Table 3:** Energy status and macronutrient proportion in men and in women during control and exercise conditions.

	Men			Women	
	Control	Exercise		Control	Exercise
Absolute EI (kcal)	2217.74±209.16	2188.02±255.30		1604.05±255.57	1654.58±457.47
Relative EI (kcal)	2217.74±209.16	1896.99±264.30*		1604.05±255.57	1434.17±442.38
Carbohydrates (%)	54.50±5.93	50.42±3.76*		53.10±4.17	52.37±4.77
Fat (%)	31.70±4.63	35.47±3.09*		32.07±3.46	33.17±4.86
Protein (%)	13.87±2.08	13.85±1.42		14.75±2.46	14.35±2.82

Mean±SD. * *P*≤0.05; significant difference from control.   EI = Energy intake; relative EI = absolute EI - exercise energy expenditure

Visual analogue scale was used to assess appetite rate. Subjects completed a daily questionnaire to rate appetite (IE hunger, fullness, desire to eat, how much food one can eat) as previously described (4). Questionnaires were completed during waking hours in the fasted state.

***Blood sample***

Blood sample was taken on the 6th day after control and exercise conditions during overnight fasting status. Acylated ghrelin and leptin were measured in plasma by enzyme–linked immunoassay (ELISA) method using commercial available kits (BioVendor Co, Czech Republic). Insulin was also measured in serum by CLIA method (Liason Co, Italy). Glucose was determined using enzymatic method (Glucose Oxidase and Peroxidase, Man Co. Tehran, Iran).

***Statistical analysis***

Results are expressed as mean ± standard deviation. Data was analyzed using the Statistical Package for the Social Sciences (SPSS) software version 19.0 for Windows (SPSS Inc, Chicago, IL, US.). All data were normally distributed. A two-factor repeated-measure ANOVA (sex × condition) was used for absolute and relative energy intake (averaged total 5 days in each condition), appetite rate (averaged total 5 days in each condition), fasting glucose and fasting concentrations for all of hormones. When sex differences did not exist, a paired t-test was used for all of variables in men and women independently. Significant differences were defined as α ≤ 0.05.

## Results

*Energy status*

Results showed that changes in average daily energy intake over the five days were not significant in both sexes between the two conditions. However, women compensated 22.92 % of energy expended in exercise by increasing energy intake while increasing 

energy expenditure did not lead to compensation for energy intake (-10.21% of EE) in men. Interestingly, relative energy intake (energy intake – exercise energy expenditure) reduced in exercise condition only in men (*P*=0.018). 

*Macronutrient*

In men, fat proportion in food intake was elevated in exercise condition (*P* =0.026). Also, carbohydrate proportion decrease significantly by exercise (*P*=0.039). However, these differences were not significant in women. There was no significant sex difference in composition of food consumed.

*Blood variables*

No significant changes were found in acylated ghrelin, leptin and glucose levels between conditions in both sexes. However, insulin concentrations decreased significantly after exercise only in women (*P*=0.007). In both conditions, fasting leptin concentrations were significantly higher in women than in men (*P* ≤0.001). 

*Appetite*

Hunger, fullness, desire to eat and prospective food consumption (PFC) did not change in either sex in response to exercise. In addition, no significant sex differences in appetite responses to exercise were observed. 

## Discussion

There is a huge body of evidence supporting that physical exercise or training can decrease adiposity, play an important role in energy expenditure, and influence hormones concentrations. Becoming motivated to eat and intake food in response to acute exercise seem to be modulated by gender, body weight and eating behavior. The evidence to date emphasizes the need to increase physical exercise levels, particularly when there is a high prevalence of obesity.

**Table 4:** Appetite rate in men and in women


	Men			Women	
	Control	Exercise		Control	Exercise
Hunger	37.52±10.61	35.77±10.27		31.40±8.58	29.22±11.48
Fullness	23.70±13.12	23.77±9.56		31.57±7.14	30.77±15.44
Desire to eat	40.57±10.40	39.35±6.73		32.25±9.40	29.37±12.30
PFC	41.77±8.54	39.27±6.59		32.30±11.41	31.95±11.48

Mean±SD. * *P*≤0.05; significant difference from control

**Figure 1:**
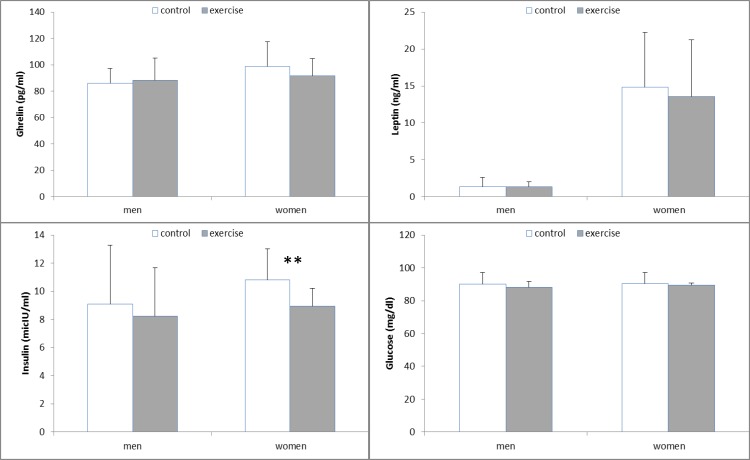
Fasting hormone and glucose concentration in men and women during control and exercise conditions.

Mean±SD. ** *P*≤0.01; significant difference from control

The main findings of this study are that low-intensity exercise for five consecutive days has no effect on absolute energy intake. Furthermore, this exercise program can cause a negative energy balance only in men. This finding is generally consistent with other previous well-controlled exercise studies (4, 5).

There is evidence suggesting that women increase their energy intake in response to exercise-induced energy deficit and maintain their body weight (1). However, men do not sufficiently raise their energy intake and experience a negative energy balance with exercise. In addition, previous studies showed that long-term aerobic exercise decreased body fat in men with no significant effect in women (2, 3). These data illustrate that women are more resistant to weight loss and there are clear sex differences in their response to exercise. In our study, energy intake compensation in response to exercise was -10 % and 23 % in men and women, respectively. However, given our relatively small sample size, we did not detect a significant sex difference in absolute energy intake response to exercise. 

In contrast with our results, Staten (11) and Whybrow *et al* (12) reported that men increased their food intake to compensate energy cost of exercise whereas women did not. Differences between the protocols may explain the disparity in results. In Staten’s study, intensity and duration of exercise were 68% VO2max and 1 h, respectively. In addition, energy cost of exercise in Whybrow *et al* (12) study was more than our study. There is not sufficient evidence in this area and further studies are needed to investigate the effect of intensity, duration and energy cost of exercise on energy balance between men and women.

Unlike our study, food intake in some previous studies was not *ad libitum* (10) and composition of diet was fixed (12). Those results cannot be generalized to real condition, because the fixed composition of the available diet restricts food and macronutrient choice. In the current study, subjects were free to eat whatever they wanted. Our results showed that men changed their food composition in response to exercise, but women did not. This finding can be related to negative energy balance. It may be concluded that men eat more fat in response to negative energy balance for getting more energy. On the other hand, eating high-fat foods provides an opportunity to increase energy intake without increasing the weight of food consumed. 

In the current investigation, ghrelin and leptin concentrations did not change in either sex in response to exercise. Effects of exercise on ghrelin and leptin concentrations are equivocal. It is reported that aerobic exercise causes ghrelin to decline (16-19), remain unchanged (20-24), or increase (25-28) and leptin to remain unchanged (29) or decline (30, 31). However, it is concluded that exercise, in the absence of weight loss, does not induce any significant increase in the plasma levels of leptin (32, 33) and ghrelin (34). In this study, the intensity and the duration of the exercise did not appear to be sufficient enough to affect the plasma levels of leptin and ghrelin. Furthermore, there were considerable inter-individual variations in concentrations of these two hormones both at control and in response to exercise. This may be one of the main reasons for the lack of significant differences between conditions.

We found that insulin concentration was reduced by exercise only in women which is the second observed piece of evidence indicating sex difference. Also, this result may explain our other finding about energy intake. Since insulin is known as suppressor of energy intake (35), reduction of insulin may be responsible for compensatory effect (non-significant) of exercise on food intake in women. This data is in agreement with other results (9, 10) about sex differences in hormonal secretion in response to exercise.

The finding that appetite rate was the same in control and exercise conditions in both sexes is in agreement with previous findings, which have shown no impact of short-term exercise on appetite (4, 12). However, Hagubian *et al* found that raising energy expenditure with exercise for four days with energy added decreases appetite rate in men but not in women (10). In addition to differences in subject’s fat percent, the most obvious difference is that food intake was not *ad libitum* in that study while in our and other studies (12); appetite responses to exercise were assessed in subjects who ate *ad libitum*. It is predictable that when food intake is *ad libitum* in two conditions (control & exercise), subjects eat enough food and feel the same appetite sensations.

This is the only study to assess the effect of short-term exercise (over several consecutive days) on all of three dependent variables (appetite, hormones and energy intake) in the same study. Also, our study conducted in a natural setting in contrast to a laboratory setting in regard to food intake. However, there are other hormones involved in body energy balance that we did not measure. Furthermore, the sample size was limited to eight men and eight women and there are considerable limitations in predicting exercise energy expenditure. Clearly, further researches are required with fewer limitations to fully investigate the probable sex differences effect on energy balance in response to exercise. 

## Conclusion

In this study, five consecutive exercise days could not cause a negative energy balance in women. It seems that women are more sensitive to negative energy balance and regulate their energy intake as well as some energy-regulating hormones in response to exercise-induced energy deficit. Further researches are needed to confidently recommend that women may need both to increase energy expenditure and decrease energy intake to loose body fat. 
